# Efficacy of immediate postoperative intravenous iron supplementation after staged bilateral total knee arthroplasty

**DOI:** 10.1186/s12891-023-06133-2

**Published:** 2023-01-07

**Authors:** Sung-Sahn Lee, Jeounghun Lee, Young-Wan Moon

**Affiliations:** 1 Department of Orthopedic Surgery, Ilsan Paik Hospital, Inje University School of Medicine, Gyeonggido Goyangsi, South Korea; 2 Department of Orthopedic Surgery, Samsung Medical Center, Sungkyunkwan University School of Medicine, 81 Ilwon street, Gangnam-Gu, 06351 Seoul, South Korea

**Keywords:** Iron supplementation, Intravenous iron, Total knee arthroplasty, Hemoglobin

## Abstract

**Background:**

Approximately 26% of patients undergoing major orthopedic elective procedures have preoperative anemia. This study aimed to investigate the effect of intravenous (IV) iron supplementation on the hemoglobin (Hb) level after staged bilateral total knee arthroplasty (TKA) in patients with or without preoperative anemia.

**Methods:**

We retrospectively analyzed 418 patients who underwent staged bilateral TKA (1 week interval). The iron group (*n* = 220) received IV iron isomaltoside immediately after each TKA. The no-iron group (*n* = 198) was recommended to receive transfusion if postoperative anemia was diagnosed between the first and second TKA. Preoperative anemia was present in 42 (21.2%) and 50 (22.7%) patients in the no-iron and iron groups, respectively. Demographic data, preoperative and postoperative Hb levels, Hb level change (preoperative minus postoperative 6-week Hb level), and blood drainage amount were compared between groups.

**Results:**

The transfusion rate was lower in the iron group than in the no-iron group (96.5% vs. 58.6%, *P* < 0.001). Overall, the demographic data, preoperative and postoperative 6-week Hb levels, Hb level change, and blood drainage amount were not significantly different between the two groups. Among patients with preoperative anemia, the iron group showed lower Hb level change (0.6 ± 0.9 vs. 0.1 ± 1.1, *P* = 0.016).

**Conclusion:**

Patients with preoperative anemia treated with IV iron showed lower Hb level change than did those without IV iron treatment. Despite the lower transfusion rate, the iron group showed similar postoperative 6-week Hb level and Hb level change to the no-iron group.

## Introduction

Total knee arthroplasty (TKA) is the gold standard treatment for patients with end-stage knee osteoarthritis. Patients undergoing TKA are typically elderly patients with multiple comorbidities. TKA is associated with substantial postoperative anemia in approximately 90% of the patients [1]. Previous studies have reported that the hemoglobin (Hb) level decreases by approximately 3 g/dL after TKA [2, 3]. The presence of moderate to severe postoperative anemia in elderly patients who have multiple comorbidities is associated with negative effects on clinical and functional outcomes [4–6]. The most effective method for the management of postoperative anemia is allogeneic blood transfusion, and previous studies have demonstrated that the rate of allogeneic transfusion ranges from 3–69% [7, 8]. However, this method has potential risks such as surgical-site infection, intravascular hemolysis, transfusion-related acute lung injury, and circulatory overload [9–12].

Iron supplementation is one option for improving postoperative Hb levels. Traditionally, iron supplementation was used only in patients with preoperative iron deficiency. However, recent studies have demonstrated that immediate postoperative iron supplementation has beneficial effects [3, 13, 14]. Intravenous (IV) iron treatment is recommended in postoperative patients because oral iron is limited by its poor absorption and frequent intolerance [15]. IV iron supplementation may lead to faster iron uptake and utilization in patients with acute postoperative blood loss [13, 14]. Several studies have demonstrated that IV iron with or without erythropoietin is safe and effective in managing postoperative anemia [16–18]. The recommended timing of IV iron initiation is several weeks before surgery; however, it has disadvantages of being time consuming and requiring repeated outpatient visits and multiple blood samplings [19]. In staged bilateral TKA, IV iron replacement after the first TKA may provide sufficient time to increase the patient’s Hb level. However, few studies have investigated the effects of immediate postoperative IV iron replacement in patients undergoing staged bilateral TKA.

Preoperative anemia is a known risk factor for delayed postoperative rehabilitation and increased complications [20, 21]. A previous study reported that approximately 26% of patients who had undergone elective orthopedic surgery had preoperative anemia [22]. Another study reported that patients with iron deficiency anemia accounted for a major proportion of patients with preoperative anemia [23]. Therefore, we believe that iron supplementation may yield different outcomes between patients with and without preoperative anemia.

The purpose of the current study was to investigate the effect of IV iron supplementation on the postoperative Hb level after staged bilateral TKA in patients with or without preoperative anemia. We hypothesized that immediate postoperative IV iron administration would result in better postoperative Hb levels after staged bilateral TKA, especially in patients with preoperative anemia.

## Materials and methods

### Patients

This was a retrospective comparative study. Data were collected from January 2019 to December 2021. Patients underwent TKA without IV iron administration from January 2019 to May 2020, whereas IV iron was administered to patients immediately after surgery from June 2020 to December 2021. Patients who (1) had undergone staged bilateral TKA (1 week interval) for osteoarthritis (Kellgren–Lawrence grade 3 or 4) or spontaneous osteonecrosis and (2) had received a specific implant system (Lospa knee system; Corentec, Seoul, Republic of Korea) were enrolled. The exclusion criteria were as follows: (1) TKA for rheumatoid arthritis or other systemic inflammatory diseases, (2) loss to follow-up within 6 weeks postoperatively, and (3) unavailable or inadequate laboratory data. After applying the exclusion criteria, 198 patients were included in the no-iron group (without IV iron administration) and 220 patients were included in the iron group (with IV iron administration). The patients in each group were divided into those with and without preoperative anemia. Anemia was defined as an Hb level of < 12 g/dL in women and < 13 g/dL in men according to the World Health Organization criteria [24].

**Fig. 1:**
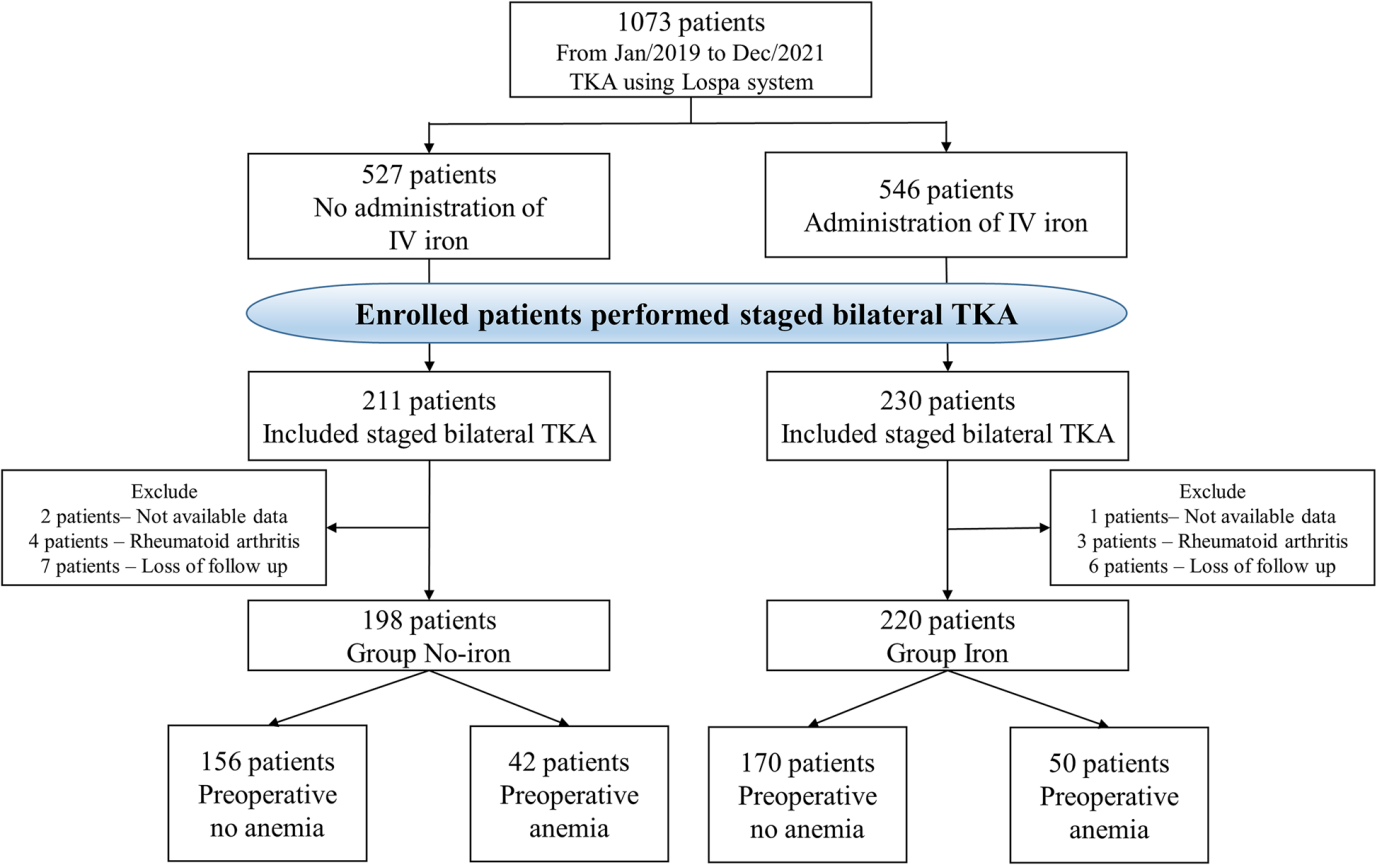
Flowchart illustrating the enrollment of patients in the current study. IV, intravenous; TKA, total knee arthroplasty

A flowchart illustrating the enrollment of patients in this study is shown in Fig. 1. All study protocols were approved by the institutional review board of our institution (SMC 2022-01-147). Informed written consent was obtained from all individual participants included in the study.

### Surgical technique, postoperative management, and iron supplementation

A single surgeon (Y.W.M) performed all TKA procedures using the same surgical technique at a single center. The interval between the first and second TKA was 1 week. Both spinal and general anesthesia were used in patients. The surgeries were performed using a tourniquet after a single dose of antibiotics. Median skin incision and medial parapatellar arthrotomy were routinely performed, whereas patellar resurfacing was not conducted. The anterior and posterior cruciate ligaments were removed, and distal femoral resection was performed using an intramedullary cutting guide. Proximal tibial cutting was performed using an extramedullary guide. Ligament balancing was performed after bone resection including the deep medial collateral ligament, posterior capsule, superficial medial collateral ligament, and pes anserinus. The Lospa knee system was implanted with cement. IV tranexamic acid of 10 mg/kg was administered to reduce bleeding, if not contraindicated [25]. Postoperative subcutaneous closed suction drainage was applied for 2 days after each TKA.

In the iron group, 400 mg IV iron isomaltoside (Monofer; Pharmacosmos A/S, Holbæk, Denmark) diluted in 200 mL normal saline was administered for 2 h immediately after each surgery. Transfusion was performed when patients had abnormal vital signs such as hypotension, tachycardia, tachypnea, hypoxia, or associated symptoms such as dyspnea or palpitation. However, in the no-iron group, transfusion was recommended when low Hb levels were detected after the first surgery, indicating anemia. Additional transfusions were administered when patients had abnormal vital signs after the second TKA.

### Outcome assessments

The patients’ demographic data, including age, sex, and body mass index, were obtained. The American Society of Anesthesiologists (ASA) classification and preoperative morbidities (e.g., hypertension, diabetes mellitus, heart disease, cerebrovascular disease, and nephrotic disease) were also assessed [26]. Preoperative and postoperative Hb levels, including the Hb level on postoperative day 5 (POD-5) after the first and second TKA and that on postoperative week 6 (POW-6) after the second TKA, were investigated. The primary outcomes were the Hb level on POW-6 and changes in the Hb level between before surgery and POW-6. The blood drainage amount (sum of the drainage amounts in the first and second TKA), transfusion rate, and postoperative periprosthetic joint infection rate were also investigated. All data were compared between the two groups. Subgroup analysis depending on the presence of preoperative anemia was also performed to determine whether IV iron administration provided different results in patients with or without preoperative anemia.

### Statistical analysis

The Shapiro–Wilk test was used to evaluate the normality of data distribution. Student’s t-test for continuous variables and the chi-squared test for categorical variables were used for comparisons between the two groups. All data were analyzed using SPSS (version 27.0; IBM, Armonk, NY, USA), and statistical significance was set at *P* < 0.05. In our study, 198 and 220 patients were allocated to the no-iron and iron groups, respectively. It would take 99% statistical power to detect a difference of at least 0.5 g/dL with a standard deviation of 1 g/dL in the postoperative Hb (α = 0.05).

## Results

With respect to demographic data, the iron and no-iron groups showed similar mean age, sex ratio, and mean body mass index. The preoperative ASA classification and preoperative morbidities, including hypertension, diabetes mellitus, heart disease, cerebrovascular disease, and nephrotic disease, were also not significantly different between the two groups (Table 1). In the subgroup analysis, patients with and without anemia had similar demographic data and preoperative morbidities (Tables 2 and 3).

**Table 1:** Comparison of demographic data and preoperative morbidities between groups (all patients)

	No-iron group	Iron group	*P*-value
No. of patients	198	220	
Age, years	70.8 ± 6.8	71.8 ± 7.4	0.143
Sex, Male:Female	30:168	42:178	0.301
Body mass index, kg/m^2^	27.5 ± 3.6	27.7 ± 3.6	0.566
ASA classification, 1/2/3/4	7/178/13/0	5/191/24/0	0.229
Hypertension, n (%)	127 (64.1%)	137 (62.3%)	0.761
Diabetes mellitus, n (%)	41 (20.7%)	52 (23.6%)	0.483
Heart disease, n (%)	12 (6.1%)	15 (6.8%)	0.843
Cerebrovascular disease, n (%)	9 (4.5%)	14 (6.4%)	0.521
Nephrotic disease, n (%)	11 (5.6%)	11 (5%)	0.829
Preoperative anemia, n (%)	42 (21.2%)	50 (22.7%)	0.724

*ASA *American Society of Anesthesiologists

**Table 2:** Comparison of demographic data and preoperative morbidities between groups (patients without preoperative anemia)

	No-iron group	Iron group	*P*-value
No. of patients	156	170	
Age, years	70.3 ± 6.5	71.1 ± 6.5	0.239
Sex, Male:Female	25:131	38:132	0.161
Body mass index, kg/m^2^	27.8 ± 3.6	28.0 ± 3.6	0.687
ASA classification, 1/2/3/4	7/142/7/0	5/151/14/0	0.324
Hypertension, n (%)	94 (60.3%)	100 (58.8%)	0.822
Diabetes mellitus, n (%)	31 (19.9%)	40 (23.5%)	0.502
Heart disease, n (%)	6 (3.8%)	8 (4.7%)	0.789
Cerebrovascular disease, n (%)	4 (2.6%)	8 (4.7%)	0.384
Nephrotic disease, n (%)	4 (2.6%)	7 (4.1%)	0.546

*ASA *American Society of Anesthesiologists

**Table 3:** Comparison of demographic data and preoperative morbidities between groups (patients with preoperative anemia)

	No-iron group	Iron group	*P*-value
No. of patients	42	50	
Age, years	72.9 ± 7.8	74.0 ± 5.6	0.426
Sex, Male:Female	5:37	4:46	0.727
Body mass index, kg/m^2^	26.6 ± 3.4	27.0 ± 3.4	0.554
ASA classification, 1/2/3/4	0/35/6/0	0/40/10/0	0.587
Hypertension, n (%)	33 (78.6%)	37 (74%)	0.634
Diabetes mellitus, n (%)	10 (23.8%)	12 (24%)	0.983
Heart disease, n (%)	6 (14.3%)	7 (14%)	0.969
Cerebrovascular disease, n (%)	5 (11.9%)	6 (12%)	0.989
Nephrotic disease, n (%)	7 (16.7%)	4 (8%)	0.334

*ASA *American Society of Anesthesiologists

Among all patients, the rate of IV tranexamic acid administration was similar between the two groups (no-iron group vs. iron group, 92.4% vs. 92.5%; *P* = 0.906). The transfusion rate was significantly higher in the no-iron group (no-iron group vs. iron group, 96.5% vs. 58.6%; *P* < 0.001) owing to the different indications for transfusion between the two groups. Because of the higher transfusion rate, the no-iron group showed significantly higher Hb level on POD-5 of the first and second TKA. However, the Hb level on POW-6 was similar between groups, and the change in Hb level was also not significantly different (Table 4). Patients without preoperative anemia showed similar results to those obtained in the overall patients: the no-iron group showed a higher transfusion rate and a higher Hb level on POD-5 of the first and second TKA, but had similar Hb level on POW-6 and Hb level change to the iron group (Table 5). However, a different result was obtained in patients with preoperative anemia: the mean change in Hb level was significantly lower in the iron group despite the lower transfusion rate (no-iron group vs. iron group, 0.6 ± 0.9 vs. 0.1 ± 1.1, *P* = 0.016; Table 6). The other variables showed similar results to those obtained in the overall patients.

**Table 4:** Comparison of hemoglobin levels, postoperative variables, and outcomes between groups (all patients)

	No-iron group	Iron group	*P*-value
Blood drainage amount, mL	854.1 ± 432.5	850.5 ± 439.0	0.934
Transfusion, n (%)	191 (96.5%)	129 (58.6%)	** < 0.001**
Use of tranexamic acid, n (%)	183 (92.4%)	204 (92.7%)	0.906
Preoperative Hb, g/dL	12.9 ± 1.2	12.9 ± 1.4	0.834
Postoperative Hb, g/dL			
First TKA POD-5	9.9 ± 1.2	9.3 ± 1.3	** < 0.001**
Second TKA POD-5	9.8 ± 1.3	9.1 ± 0.9	** < 0.001**
Second TKA POW-6	12.0 ± 1.0	11.9 ± 1.3	0.765
Change in Hb level (preoperative – POW-6), g/dL	1.0 ± 0.9	1.0 ± 1.4	0.722
Infection rate, n (%)	2 (1%)	0 (0%)	0.224

*Hb* hemoglobin, *TKA* total knee arthroplasty, *POD* postoperative day, *POW* postoperative week

**Table 5:** Comparison of hemoglobin levels, postoperative variables, and outcomes between groups (patients without preoperative anemia)

	No-iron group	Iron group	*P*-value
Blood drainage amount, mL	839.9 ± 442.3	871.9 ± 455.9	0.522
Transfusion, n (%)	150 (96.2%)	88 (51.8%)	** < 0.001**
Use of tranexamic acid, n (%)	150 (96.2%)	160 (94.1%)	0.45
Preoperative Hb, g/dL	13.3 ± 0.9	13.5 ± 1.0	0.163
Postoperative Hb, g/dL			
First TKA POD-5	10.0 ± 1.2	9.6 ± 1.3	** < 0.001**
Second TKA POD-5	10.0 ± 1.3	9.3 ± 0.9	** < 0.001**
Second TKA POW-6	12.2 ± 0.9	12.2 ± 1.3	0.595
Change in Hb level (preoperative – POW-6), g/dL	1.1 ± 0.9	1.3 ± 1.3	0.079
Infection rate, n (%)	0 (0%)	0 (0%)	1

*Hb* hemoglobin, *TKA* total knee arthroplasty, *POD* postoperative day, *POW* postoperative week

**Table 6:** Comparison of hemoglobin levels, postoperative variables, and outcomes between groups (patients with preoperative anemia)

	No-iron group	Iron group	*P*-value
Blood drainage amount, mL	906.4 ± 394.5	777.8 ± 370.6	0.111
Transfusion, n (%)	41 (97.6%)	41 (82%)	**0.019**
Use of tranexamic acid, n (%)	33 (78.6%)	44 (88%)	0.265
Preoperative Hb, g/dL	11.3 ± 0.8	11.1 ± 0.8	0.125
Postoperative Hb, g/dL			
First TKA POD-5	9.2 ± 0.9	8.3 ± 0.8	** < 0.001**
Second TKA POD-5	9.1 ± 0.8	8.6 ± 0.7	**0.005**
Second TKA POW-6	10.8 ± 0.6	11.0 ± 0.9	0.313
Change in Hb level (preoperative – POW-6), g/dL	0.6 ± 0.9	0.1 ± 1.1	**0.016**
Infection rate, n (%)	2 (4.8%)	0 (0%)	0.206

*Hb* hemoglobin *TKA* total knee arthroplasty, *POD* postoperative day, *POW* postoperative week

In terms of postoperative complications, periprosthetic joint infection occurred in two patients with preoperative anemia in the no-iron group (*P* = 0.206, Table 6). These patients were readmitted and treated with open debridement and IV antibiotics.

## Discussion

The principal finding of the present study was that IV iron administration aided the recovery of Hb level until POW-6 in patients with preoperative anemia who had undergone staged bilateral TKA. Despite their lower transfusion rate, patients who had received IV iron showed similar Hb levels on POW-6 to those who did not receive IV iron treatment.

A surgery-induced inflammatory response may lead to a functional iron deficiency, similar to anemia development due to chronic diseases [27]. Increased hepcidin levels due to inflammation may play a major role in the development of functional iron deficiency, which can last for several weeks postoperatively [15]. Moreover, surgical bleeding directly induces iron loss. A previous study reported that > 1 month is needed for Hb recovery after 500 mL of whole blood donation. Therefore, iron supplementation can help replace the depleted iron stores due to surgical bleeding, especially in patients undergoing TKA, which is expected to result in a blood loss of > 500 mL [28, 29]. On the basis of this evidence, many studies have investigated the efficacy of IV iron supplementation in patients undergoing TKA [3, 13, 14]. Recent meta-analysis studies demonstrated that iron supplementation significantly reduced the transfusion rate and increased the postoperative Hb level in patients who underwent unilateral TKA [13, 14]. In our study, the transfusion rate was lower in the iron group because of the different indications for transfusion between the two groups. Therefore, comparing the transfusion rate is not meaningful. The Hb level on POW-6 and the change in Hb levels did not differ between the two groups in all patient comparisons. However, in the subgroup analysis of patients with preoperative anemia, the change in Hb level (preoperative Hb level minus POW-6 Hb level) was significantly lower in patients with iron supplementation. A previous study reported that 26% of patients who underwent elective orthopedic surgery had preoperative anemia [22]. The prevalence of iron deficiency anemia in non-hospitalized older adults is 16.6–25%; therefore, many patients who are scheduled for TKA may have preoperative iron deficiency anemia [23]. Preoperative anemia is a major risk factor for 30-day complications, including infection, cardiac complications, and prolonged hospital stay [20, 21, 30]. A preoperative Hb level of < 12.5 g/dL was associated with postoperative transfusion after TKA [20]. Therefore, blood management is more necessary in patients with preoperative anemia than in those without. Our results support the hypothesis that immediate postoperative IV iron supplementation may be beneficial in patients scheduled to undergo staged bilateral TKA in terms of reducing the need for blood management in those with preoperative anemia.

Patients who plan to undergo TKA in both knees can choose between simultaneous and staged bilateral TKA. If staged bilateral TKA is selected, the interval between the two surgeries must be decided. Previous studies demonstrated that the interval between the two TKA procedures did not influence the medical or surgical complications after the second surgery, and the clinical and functional results did not differ depending on the timing of the second TKA [31, 32]. Although many studies have investigated the effect of IV iron administration in patients undergoing unilateral TKA, few studies have explored the correlation between IV iron treatment and staged bilateral TKA. In staged bilateral TKA, IV iron replacement after the first TKA can provide sufficient time to increase the patient’s Hb level; therefore, it was hypothesized that IV iron replacement would yield better results in terms of transfusion rate and postoperative Hb level in staged bilateral TKA than in unilateral TKA Jeong et al. [30] compared the transfusion rate and postoperative Hb levels between the IV iron supplementation group (*n* = 65) and the no supplementation group (*n* = 61) in patients who underwent staged bilateral TKA. They concluded that IV iron had no effect on the transfusion rate or the postoperative Hb level. The average lifespan of red blood cells is approximately 120 days, whereas that of transfused red blood cells is approximately 60 days [33]. In our study, similar results were obtained for postoperative Hb level in all patients. As different transfusion rates can affect the Hb level on POW-6, the iron group can be expected to show higher Hb levels on POW-6 than the no-iron group if they have a similar transfusion rate.

The current study had several limitations. First, the indication for transfusion differed between the two groups. Approximately 96.5% of patients in the no-iron group received allogeneic blood transfusion, whereas only 58.6% in the iron group received allogeneic transfusion. Considering the lifespan of transfused red blood cells, the Hb levels on POW-6 might have been affected by transfusion. If the transfusion rate was similar between the two groups, the Hb level on POW-6 might have been lower and the change in Hb level might have been greater in the no-iron group. In patients with preoperative anemia, the change in Hb level was significantly lower in the iron group despite the lower transfusion rate. If the transfusion rate was similar among patients with preoperative anemia, the difference in the change in Hb level between the two groups would have been greater. Therefore, we believe that the different indications for transfusion between the two groups would not influence our findings in patients with preoperative anemia. Second, the iron profile parameters, including serum iron, ferritin, total iron-binding capacity, and transferrin saturation, were not evaluated. In particular, the preoperative iron profile was not available. Therefore, it was difficult to determine the proportion of patients with iron deficiency anemia or those with postoperative improvements in iron profile parameters among patients with preoperative anemia. Third, the effect of postoperative IV iron administration may differ according to the dose of iron. It was poorly investigated that optimal dosage of iron supplement. Some previous studies used more than 1 g iron isomaltoside for experimental group. However, a previous study demonstrated that the administration of 300 mg iron sucrose caused serum iron overload on POD-1 [30]. As the iron content of iron isomaltoside is four-thirds that of iron sucrose, we believe that our study would have shown iron overload similar to that in the previous study [34]. Fourth, this study had inherent limitations due to its retrospective, nonrandomized design. A randomized controlled study with iron profile data should be conducted to overcome the limitations of our study. Fifth, Laboratory tests including Hb were not performed between POD-5 and POW-6. So, it is a major disadvantage that Hb level at other times were not able to assess.

## Conclusion

Patients with preoperative anemia treated with IV iron showed a lower change in Hb level than did those without IV iron treatment. Despite the lower transfusion rate, patients with immediate postoperative IV iron administration showed similar Hb levels on POW-6 and Hb level change to those who did not receive postoperative IV iron replacement.

## Data Availability

The datasets used and/or analyzed during the current study available from the corresponding author on reasonable request.

## Abbreviations

ASA: American Society of Anesthesiologists
Hb: Hemoglobin
IV: Intravenous
POD: Postoperative day
POW: Postoperative week
TKA: Total knee arthroplasty
